# Aspirin mediates histone methylation that inhibits inflammation-related stemness gene expression to diminish cancer stemness via COX-independent manner

**DOI:** 10.1186/s13287-020-01884-4

**Published:** 2020-08-27

**Authors:** Xiaoyuan Zhang, Renle Du, Na Luo, Rong Xiang, Wenzhi Shen

**Affiliations:** 1 Department of Pathology and Institute of Precision Medicine, Jining Medical University, 133 Hehua Road, Jining, 272067 China; 2 Department of Immunology, School of Medicine, Nankai University, Tianjin, 300071 China; 3 2011 Project Collaborative Innovation Center for Biotherapy of Ministry of Education, 94 Weijin Road, Tianjin, 300071 China

**Keywords:** Aspirin, Histone methylation, Cancer stemness, ICAM3, COX, Therapeutic strategies

## Abstract

**Background:**

The widely recognized anti-cancer potential of aspirin has created a broad interest to explore the clinical benefits of aspirin in cancer therapy. However, the current understanding of the molecular mechanisms involved in the anti-cancer potential of aspirin remains limited.

**Methods:**

Cancer stemness assays which contained ALDH, side population, chemo-resistance, sphere formation, and tumorigenesis were performed to validate aspirin function in vitro and in vivo. Histone modification assay was performed to check the effect of aspirin on histone methylation as well as the activity of HDAC and KDM6A/B. Inhibitor in vivo assay was performed to evaluate therapeutic effects of various inhibitor combination manners.

**Results:**

In regards to in vitro studies, aspirin diminishes cancer cell stemness properties which include reducing the ALDH+ subpopulation, side population, chemo-resistance, and sphere formation in three cancer types. In regards to in vivo studies, aspirin decreases tumor growth and metastasis and prolongs survival. In addition, our results showed that aspirin inhibits inflammation-related stemness gene expression (especially ICAM3) identified by a high-throughput siRNA platform. In regards to the underlying molecular mechanism of action, aspirin reduces histone demethylase (KDM6A/B) expression that mediates histone methylation and suppresses gene expression via a COX-independent manner. In regards to therapeutic strategies, aspirin combined HDM inhibitors, ICAM3 downstream signaling Src/PI3K inhibitors, or ICAM3 inhibitor Lifitigrast prevents cancer progression in vivo.

**Conclusions:**

The aforementioned findings suggest a molecular model that explains how aspirin diminishes cancer cell stemness properties. These findings may provide novel targets for therapeutic strategies involving aspirin in the prevention of cancer progression.

## Background

Cancer stem cell (CSC) was found as the chief culprit to initiate tumor occurrence, to enhance tumor malignancy and to cause tumor recurrence, whereby the maintenance of cancer cell stemness mainly depends on the tumor micro-environment or also called the “niche” [1–3]. Currently, the specific properties of CSC were identified like high ALDH1 activity (aldehyde dehydrogenase 1) [4], side population [5], chemo-resistance, and other CD molecules (CD44, CD133) or markers (SOX2, OCT4, NANOG, LGR5) positive in cancer. Clinical therapy which targeted highly tumorigenic CSCs may provide new targets or insight for cancer therapy; however, unfortunately, CSCs had demonstrated a relative resistance to conventional chemotherapy and radiotherapy. Moreover, the cancer cell stemness and the resultant tumor initiation/malignancy could be maintained by the tumor-associated inflammation factors within the tumor micro-environment (niche) [6, 7]. Our previous work presented a medium-throughput siRNA screen platform to identify inflammation genes that regulate cancer cell stemness and obtained several novel candidates [8]. Agents that target these genes may inhibit both inflammation and cancer cell stemness at the same time.

Aspirin (a non-steroidal anti-inflammatory drug) is commonly used as an antipyretic, analgesic, anti-inflammatory, and anti-thrombotic agent [9, 10]. Recent observational and epidemiological studies have shown that regular, prolonged use of aspirin reduces the risk for several cancers (e.g., colorectal, esophageal, breast, lung, prostate, liver, and skin cancers) [11–13]. Although the benefits of aspirin for cancer patients have been widely appreciated, the mechanism remains unclear. Previous studies attribute the anti-cancer potential of aspirin to the inhibition of cyclooxygenase-2 (COX-2) which is upregulated in various cancer cells [14, 15]. Of note, an increasing body of evidence suggests that aspirin exhibits anti-cancer effects in a COX-independent manner [16].

Histone modification is a reversible process mediated by the epigenetic enzymes [17, 18]. Histone methylation and acetylation are two important chemical modifications that act in transcriptional activation or inactivation, chromosome packaging, and DNA damage/repair [19, 20]. Histone demethylases (HDMs) and histone deacetylases (HDACs) are the key enzymes that remove methyl and acetyl groups respectively to regulate gene transcription. In this regard, aspirin was reported to affect HDACs expression and suppress progression of some cancers like aspirin mediates H3K27 acetylation to prevent colon carcinogenesis and aspirin cooperates with p300 to activate H3K9 acetylation further to promote colorectal cancer cell apoptosis [16, 21, 22]. However, the specific roles and mechanisms of aspirin-mediated histone methylation in cancer stemness remains insufficient. Thus, we studied the role of aspirin on histone methylation and the attendant effects on cancer cell stemness and cancer progression.

Our results indicated that (1) aspirin diminishes various cancer cell stemness properties which include reducing the ALDH+ subpopulation, side population, chemo-resistance, and sphere formation in three cancer types in vitro; (2) aspirin inhibits tumor growth and metastasis as well as prolongs survival in vivo; (3) aspirin inhibits inflammation-related stemness genes especially ICAM3; (4) aspirin reduces histone demethylase (KDM6A/B) expression which mediates histone 3 methylation respectively with a COX-independent manner; and (5) aspirin + HDM inhibitors, aspirin + ICAM3 downstream signaling Src/PI3K inhibitors, and aspirin + ICAM3 inhibitor Lifitigrast all reduce cancer progression in vivo. The abovementioned findings demonstrate a promising mechanism of action and potential therapeutic strategy of aspirin in the prevention of cancer progression.

## Methods

### Cell culture

MDA-MB-231 breast cancer cell and A549 lung cancer cell were purchased from ATCC; HepG2 was obtained from the Chinese Academy of Sciences. MDA-MB-231 breast cancer cell and HepG2 liver cancer cell were cultured in DMEM medium. A549 lung cancer cell was cultured in 1640 medium. All culture media were supplemented with 10% FBS (Gibco) and were grown at 37 °C in 5% CO2 incubators. All cells were passaged for less than 3 months before renewal from frozen, early-passage stocks and tested to ensure that they were Mycoplasma negative.

### Cytotoxicity assay

Aspirin was purchased in Sigma (cat. A2093) and dissolved in DMSO. MDA-MB-231, A549, and HepG2 cells were cultured in 96-well plate and treated with various concentrations (1.0, 2.0, 4.0, 8.0, 10.0, 12.0, 16.0, 20.0, and 30.0 mM for A549 cells; 0.5, 1.0, 1.5, 2.0, 4.0, 5.0, 6.0, 8.0, and 10.0 mM for MDA-MB-231 cells; and 0.5, 1.0, 2.0, 4.0, 8.0, 12.0, 16.0, 20.0, and 30.0 mM for HepG2 cells) of aspirin for 24 h. Cell activity was tested by applying CCK8 kit (Dojindo, China) following the manufacturer’s instructions.

### Aldefluor assay

The Aldeflour assay kit (STEMCELL Technologies, Vancouver, Canada) was used to measure ALDH enzymatic activity in three cancer cell lines (MDA-MB-231, A549, and HepG2). In brief, cells were treated with aspirin for 24 h, and 2.5 × 10^5^ cells were suspended in Aldeflour assay buffer containing ALDH1 substrate and incubated for 60 min at 37 °C. Cells treated with the specific ALDH inhibitor DEAB served as the negative control. Stained cells were analyzed on BD FACS Calibur flow cytometer (BD Biosciences, San Jose, CA). Data analysis was performed using Flowjo software (Tree Star, Inc., Ashland, OR).

### Side population assay

MDA-MB-231, A549, and HepG2 cells treated with aspirin for 24 h were harvested and resuspended in pre-warmed staining buffer (PBS buffer added 2% FBS) at a density of 1.0 × 10^6^ cells/ml. Hoechst 33342 dye was added at a final concentration of 7 μg/ml (231), 8 μg/ml (A549), and 10 μg/ml (HepG2) in the presence or absence of 10 μM fumitremorgin C (FTC). The following steps were described previously [8, 23].

### Cell apoptosis assay

The MDA-MB-231, A549, and HepG2 cells were treated with aspirin and cisplatin (DDP) (10 μg/ml) for 24 h simultaneously, then harvested and resuspended in pre-warmed staining buffer (PBS buffer added 2% FBS) at a density of 1.0 × 10^6^ cells/ml. Apoptotic cells were stained with propidiumiodide and Annexin-V-FITC (BD Biosciences). Flow cytometry analysis was performed by FACS Calibur cytometer (BD Biosciences), in which a minimum of 10, 000 events were recorded.

### Sphere formation assay

Cells were collected and rinsed to remove serum, then dissociated to single-cell suspension in serum-free DMEM/F12 medium supplemented with 100 IU/ml penicillin, 100 μg/ml streptomycin, 20 ng/ml human recombinant epidermal growth factor (hREGF), 20 ng/ml human recombinant basic fibroblast growth factor (bFGF), and 2% B27 supplement (Invitrogen). Cells were subsequently cultured in ultra-low attachment 24-well plates at a density of 500 cells per well [8], then treated with aspirin all the time until the spheres were formed.

### Animal study

Female Balb/c mice at 6–8 weeks were separated randomly into several groups (*n* ≥ 5). 5 × 10^4^ 4T1-luci cells were inoculated s.c into each mouse at the right axilla. For lung metastasis assay, at 7 days after injection, mice were treated intraperitoneally with aspirin 50 mg/kg, aspirin 100 mg/kg every 3 days, and DMSO used as the control. For chemo-resistance assay, at 7 days after injection, mice were first treated intraperitoneally with cisplatin (2.5 mg/kg), then treated with aspirin 25 mg/kg, aspirin 50 mg/kg every 3 days, and DMSO used as the control.

NOD/SCID mice at 6–8 weeks were separated randomly into several groups (*n* ≥ 5). 3 × 10^6^ A549-luci cells were inoculated s.c into each mouse. For inhibitor treatment assay, 23 days after tumor cells injection, mice were first treated intraperitoneally with aspirin 25 mg/kg or KDM6A/B inhibitor (GSK J1, 100 mg/kg) or Src inhibitor (a new specific high-efficient Src inhibitor [8, 24], 20 mg/kg) or PI3K inhibitor (LY294002, 70 mg/kg) or Lifitegrast (20 mg/kg). DMSO used as the control.

Tumor volume (mm^3^) was measured with calipers and calculated by using the standard formula: length × width^2^/2. The individual measuring the mice was unaware of the identity of the group measured. Primary tumor tissues were harvested and separated into three parts; one was formalin fixed, paraffin embedded, and sectioned for IHC staining, the other two were broken by the tissue homogenizer, then used for RNA (TRIzol) and protein (RIPA lysis buffer) extraction. Animal use complied with Nankai University and Jining Medical University Animal Welfare Guidelines [25].

### Western blotting

The western blot steps were described previously [8, 26]. The special primary antibodies used in this assay are listed in supplementary Table S1. All western data was the representative image of three biologically independent repeats. The results were quantified using Image J software (National Institutes of Health, Baltimore, MD) and analyzed by Graphpad Prism5 software (GraphPad Software, San Diego, CA, USA).

### Nuclear fractionation analysis

Cells were harvested, and the cytoplasmic and nuclear fractions were separated and extracted with an NE-PER Nuclear and Cytoplasmic Extraction Kit (Thermo Fisher Scientific Inc. MA, USA). H3K-3Me marker proteins were detected by western blot.

### Immunofluorescence

Cells grow on glass slides, and tumor tissue slices were fixed in 4% paraformaldehyde and labeled with primary antibodies overnight at 4 °C, followed by incubation with species-appropriate secondary antibodies at room temperature for 1 h. Nuclei were stained with DAPI, and images were obtained using a Leica DM4000 upright microscope or confocal fluorescence microscopy (Nikon, Tokyo, Japan) [8, 25].

### Chromatin immunoprecipitation assay

The assay was performed with an EZ-Zyme Chromatin Prep Kit (Millipore), according to the manufacturer’s protocol. Anti-histone 3 modification marker antibodies were used to precipitate DNA cross-linked with histone 3 modification markers, respectively, and normal rabbit IgG was used in parallel as a control. Enriched DNA was then used as a template to assess the binding intensity of histone 3 modification markers to putative binding sites in the ICAM3 promoter. Primers used in this assay are listed in supplementary Table S2.

### Immunohistochemistry

Immunohistochemistry was performed on tumor tissue sections from the mice. Primary antibodies raise against the target proteins at a 1:100 dilution overnight. The expression levels of the proteins were evaluated according to the percentage of positive cells in each tumor tissue sections. The images were recorded by Olympus BX51 Epi-fluorescent microscopy under a × 20 or × 40 objective (Olympus Co., Tokyo, Japan) [27].

### Statistical analysis

All data were analyzed using GraphPad Prism5 software (GraphPad Software, San Diego, CA, USA). Values were expressed as means ± SEM. *P* values were calculated using a two-tailed Student’s *t* test (two groups) or one-way ANOVA (more than 2 groups) unless otherwise noted. A value of *P* < 0.05 was used as the criterion for statistical significance. An asterisk indicates a significant difference with *P* < 0.05, two asterisks indicate a significant difference with *P* < 0.01, and three asterisks indicate a significant difference with *P* < 0.001 [8, 28].

## Results

### Aspirin diminishes cancer cell stemness properties in vitro

In order to establish the proper working concentrations of aspirin in various cancer cells, we determined the IC50 of aspirin in A549 lung cancer cells, MDA-MB-231 breast cancer cells, and HepG2 liver cancer cells using a cytotoxicity assay. Our results showed a 10.7-mM IC50 in A549 lung cancer cells, a 4.3-mM IC50 in MDA-MB-231 breast cancer cells, and a 9.7-mM IC50 in HepG2 liver cancer cells (Fig. S1). Based on the IC50, we chose working concentrations of 0, 5, and 10 mM aspirin for A549 lung cancer cells; 0, 2, and 4 mM aspirin for MDA-MB-231 breast cancer cells; and 0, 5, and 10 mM aspirin for HepG2 liver cancer cells in our studies.

**Fig. 1:**
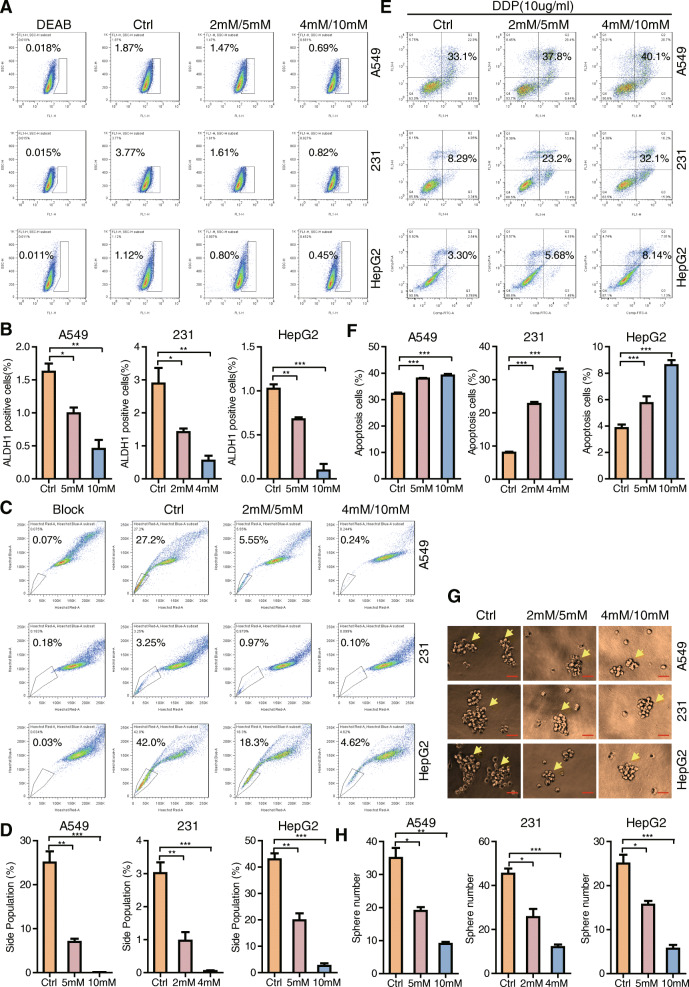
Aspirin restrains cancer cell stemness properties in vitro. **a** ALDH staining assay was performed to check ALDH+ sub-population percentage in the three cancer cell lines with or without aspirin treatment. **b** Statistic results of ALDH sub-population percentage were shown. **c** Side population assay was performed to detect SP percentage in three cancer cell lines with or without aspirin treatment. **d** Statistic results of SP percentage were shown. **e** FACS was performed to detect cell resistance to cisplatin, and the percentage of apoptotic cells was shown. **f** Statistic results of apoptosis cells percentage were shown. **g** Sphere formation assay was performed to check the cell sphere formation ability in the three cancer cell lines with or without aspirin treatment. Scale bars, 100 μm. **h** Statistic results of sphere amounts were shown

In order to determine the in vitro effects of aspirin on cancer cell stemness, we investigated ALDH+ sub-population changes in A549 lung cancer cells, MDA-MB-231 breast cancer cells, and HepG2 liver cancer cells using the ALDH staining assay. Our results indicated that the ALDH+ sub-population decreases in the aspirin-treated groups versus controls (Fig. 1a, b). In order to determine the effects of aspirin on cancer cell stemness, we next investigated the changes in the side-population in the three cancer cell lines using the side population assay. Our results indicated that the side population decreases in the aspirin-treated groups versus controls (Fig. 1c, d). In order to determine the effects of aspirin on cancer cell stemness, we next investigated the changes in apoptosis in the three cancer cell lines using the cell apoptosis assay after cisplatin (DDP) treatment. Our results indicated that apoptosis increases in the aspirin-treated groups versus controls and thereby reduces cisplatin- (chemo-) resistance (Fig. 1e, f). In order to determine the effects of aspirin on cancer cell stemness, we next investigated the changes in cell sphere formation in the three cancer cell lines using the sphere formation assay. Our results showed that sphere formation decreases in the aspirin-treated groups versus controls (Fig. 1g, h). The abovementioned findings suggest that aspirin diminishes cancer cell stemness properties in vitro.

### Aspirin diminishes cancer cell metastasis and stemness properties in vivo

**Fig. 2:**
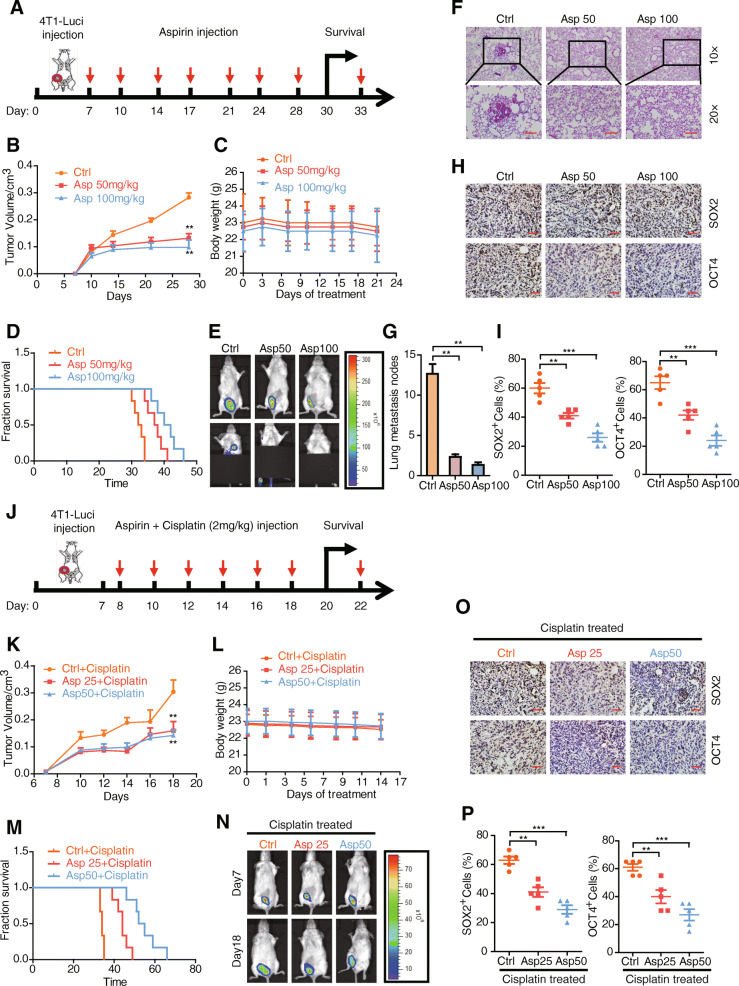
Aspirin suppresses cancer cell metastasis and stemness in vivo. **a** Schema of the metastasis model established by subcutaneous implantation of 4T1-luci cells into the 4th pair of mammary fat pad of BALB/c mice. **b** Tumor growth curve of 4T1-luci with or without aspirin treatment. **c** The body weight of BALB/c mice in the course of aspirin treatment. **d** The survival curve of BALB/c mice inoculated with 4T1-luci with or without aspirin treatment. **e** The representative luciferase images showing the 4T1-luci tumors at the primary site and lung metastasis sites with or without aspirin treatment. **f** Representative H&E staining images of 4T1-luci tumors metastasis to the lung with or without aspirin treatment. Scale bars (lower panel), 100 μm. **g** Statistic results of metastasis loci of 4T1-luci tumors metastasis to the lung with or without aspirin treatment. **h** Immunohistochemistry staining of SOX2 and OCT4 in 4T1-luci primary tumors with or without aspirin treatment. Representative images with × 20 magnification were shown. Scale bars, 100 μm. **i** Statistic results of SOX2 or OCT4 positive cells in 4T1-luci primary tumors with or without aspirin treatment. **j** Schema of the chemo-resistance model established by subcutaneous implantation of 4T1-luci cells into the 4th pair of mammary fat pad of BALB/c mice. **k** Tumor growth curve of 4T1-luci with or without aspirin treatment in the presence of cisplatin. **l** The body weight of BALB/c mice in the course of aspirin treatment in the presence of cisplatin. **m** The survival curve of BALB/c mice inoculated with 4T1-luci with or without aspirin treatment in the presence of cisplatin. **n** The representative luciferase images showing 4T1-luci tumors at the primary sites with or without aspirin treatment on day 7 (before cisplatin administration) and day 18 (after cisplatin administration). **o** Immunohistochemistry staining of SOX2 and OCT4 in 4T1-luci primary tumors with or without aspirin treatment in the presence of cisplatin. Representative images with × 20 magnification were shown. Scale bars, 100 μm. **p** Statistic results of SOX2 or OCT4 positive cells in 4T1-luci primary tumors with or without aspirin treatment in the presence of cisplatin

In order to determine the effects of aspirin on cancer cell metastasis and stemness in vivo, we implanted 4T1-luciferase cells into the fourth fat pad of female Balb/c mice. Seven days after implantation, we IP injected the mice with 50 mg/kg aspirin, 100 mg/kg aspirin, or DMSO (control group) 2 times per week (Fig. 2a). Our results showed that tumor volume decreases in the aspirin-treated groups versus the control (Fig. 2b). However, we found that the body weight did not change in the aspirin-treated groups versus the control (Fig. 2c). In addition, we found that the survival time increases in the aspirin-treated groups versus control (Fig. 2d). With respect to the effect of aspirin on cancer cell metastasis, we found that lung metastasis decreases in aspirin-treated groups versus the control (Fig. 2e–g). With respect to the effect of aspirin on cancer cell stemness properties, we found that the immunocytochemical staining of SOX2 and OCT4 stemness markers decreases in the aspirin-treated groups versus DMSO controls (Fig. 2h, i). The abovementioned findings suggest that aspirin diminishes cancer cell metastasis and stemness properties in vivo.

### Aspirin reduces cancer cell chemo-resistance in vivo

In order to determine the effects of aspirin on cancer cell chemo-resistance in vivo, we implanted 4T1-luciferase cells into the fourth fat pad of female Balb/c mice. Eight days after implantation, we IP injected the mice with 2 mg/kg cisplatin + 25 mg/kg aspirin, 2 mg/kg cisplatin + 50 mg/kg aspirin, or DMSO (control group) every 2 days (Fig. 2j). Our results showed that tumor volume decreases in the cisplatin/aspirin-treated versus the DMSO control (Fig. 2k). However, we found that the body weight did not change in the cisplatin/aspirin-treated versus the DMSO control (Fig. 2l). In addition, we found that the survival time increases in the cisplatin/aspirin-treated groups versus the DMSO control (Fig. 2m). We also found that the rate of tumor growth was slower in the cisplatin/aspirin-treated versus the DMSO control (Fig. 2n). With respect to the effect of aspirin on cancer cell stemness properties, we found that the immunocytochemical staining of SOX2 and OCT4 stemness markers decreases in the cisplatin/aspirin-treated groups versus DMSO controls (Fig. 2o, p). The abovementioned findings suggest that aspirin reduces cancer cell chemo-resistance in vivo.

### Aspirin inhibits the expression of inflammation-related stemness genes in vitro and in vivo

**Fig. 3:**
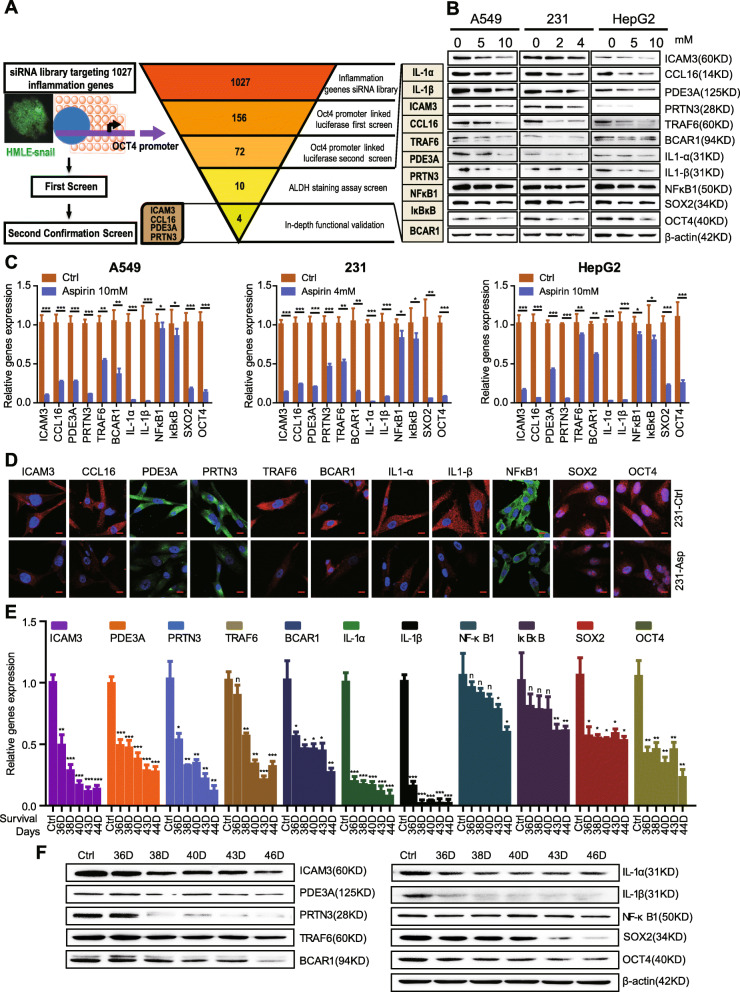
Aspirin inhibits the expression of inflammation-related stemness genes in vitro and in vivo. **a** Schematic representation of the siRNA screen (left). Summary of the results from the RNAi screen (right). **b** Western blot examining the expression of inflammatory candidates and stemness proteins (SOX2, OCT4) in A549, MDA-MB-231, and HepG2 cells with or without aspirin treatment. **c** Quantitative PCR examining the mRNA expression of inflammatory candidates and stemness genes (SOX2, OCT4) in A549, MDA-MB-231, and HepG2 cells with or without aspirin treatment. **d** Immunofluorescence staining of inflammatory candidates and stemness genes (SOX2, OCT4) in A549, MDA-MB-231, and HepG2 cells with or without aspirin treatment. Scale bars, 10 μm. **e** Quantitative PCR examining the mRNA expression of inflammatory candidates and stemness genes (SOX2, OCT4) in 4T1-luci tumors separated from BALB/c mice treated with aspirin for different survival days. **f** Western blot examining the expression of inflammatory candidates and stemness genes (SOX2, OCT4) in 4T1-luci tumors separated from BALB/c mice treated with aspirin for different survival days

Our previously published report established a medium-throughput siRNA screening platform that identifies inflammation genes that regulate cancer cell stemness [6]. Specifically, we identified several novel candidate genes that decrease OCT4 expression and the ALDH+ subpopulation both of which characterize stemness (Fig. 3a).

In order to determine whether aspirin decreases the expression of these novel candidate genes to further diminish cancer cell stemness, we investigated the expression of novel candidate genes and stemness markers (SOX2 and OCT4) in A549 lung cancer cells, MDA-MB-231 breast cancer cells, and HepG2 liver cancer cells using Western blot. Our results showed that ICAM3, CCL16, PDE3A, PRTN3, SOX2, and OCT4 protein expression decreases in the aspirin-treated groups versus controls (Fig. 3b, Fig. S2A). We also found that ICAM3, CCL16, PDE3A, PRTN3,TRAF6, BCAR1, IL-1a, IL-1b,NFkB1, IkBkB, SOX2, and OCT4 mRNA expression decreases in the aspirin-treated group versus control (Fig. 3c). Moreover, in ICAM3, CCL16, PDE3A, PRTN3, TRAF6, BCAR1, IL-1a, IL-1b, NFkB1, SOX2, and OCT4, the protein expression decreases as indicated by immunofluorescence-staining in the aspirin-treated MBA-MD-231 (Fig. 3d) and A549 cells (data not shown) versus the control.

In order to confirm the above in vitro results, we then investigated mRNA and protein expression in tumors from 36-, 38-, 40-, 43-, and 46-day aspirin-treated mice versus control using QPCR and western blot. Our results demonstrated that ICAM3, PDE3A, PRTN3, TRAF6, BCAR1, IL-1a, IL-1b, NFkB1, IkBkB, SOX2, and OCT4 mRNA expression decreases in the aspirin-treated groups versus control (Fig. 3e). In addition, we found that ICAM3, PDE3A, PRTN3, TRAF6, BCAR1, IL-1a, IL-1b, NFkB1, SOX2, and OCT4 protein expression similarly decreases in the aspirin-treated groups versus control (Fig. 3f, Fig. S2B). The abovementioned findings suggest that aspirin decreases the expression of inflammation-related stemness genes in vitro and in vivo.

### Aspirin mediates histone 3 methylation to regulate target genes expression

**Fig. 4:**
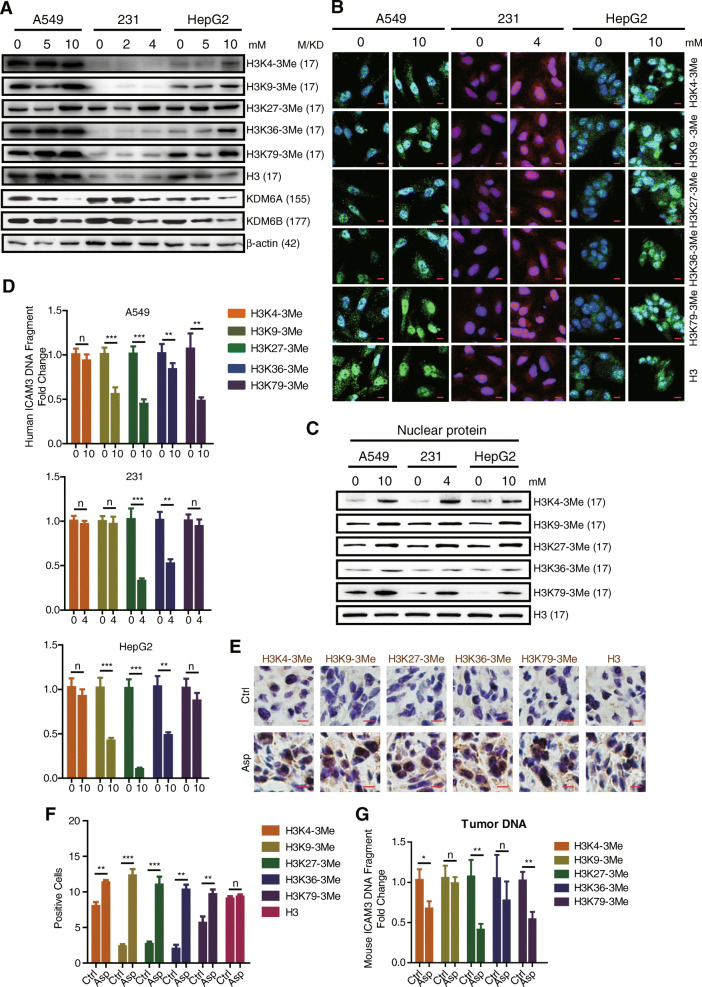
Aspirin mediates histone 3 methylation to effect target genes expression in vitro and in vivo. **a** Western blot examining the expression of histone 3 methylation markers, KDM6A, KDM6B in A549, MDA-MB-231, and HepG2 cells with or without aspirin treatment. **b** Immunofluorescence staining of H3 methylation markers in A549, MDA-MB-231, and HepG2 cells with or without aspirin treatment. Scale bars, 10 μm. **c** Western blot to check the expression of H3-3Me markers in nuclear proteins of each groups. **d** CHIP-qPCR detecting the expression of ICAM3 DNA fragment in A549, MDA-MB-231, and HepG2 cells with or without aspirin treatment. **e** Immunohistochemistry staining examining the expression of H3 methylation markers in 4T1-luci tumors separated from BALB/c mice. Representative images with × 40 magnification were shown. Scale bars, 50 μm. **f** The bar graph shows the statistic results of H3 methylation markers positive cells. **g** CHIP-qPCR detecting the expression of ICAM3 DNA fragment in 4T1-luci tumors separated from BALB/c mice with or without aspirin treatment

In order to determine the mechanism underlying the action of aspirin, we explored the regulatory effect of aspirin on histone 3 methylation markers in A549 lung cancer cells, MDA-MB-231 breast cancer cells, and HepG2 liver cancer cells using western blot. Our results indicated that the expression of H3 tri-methylation markers (i.e., H3K4-3Me, H3K9-3Me, H3K27-3Me, H3K36-3Me, and H3K79-3Me) increases in the aspirin-treated higher concentration groups versus control (Fig. 4a, S3A). We also found that the expression of histone demethylases (i.e., KDM6A and KDM6B) decreases in the aspirin-treated higher concentration groups versus control (Fig. 4a). In addition, we studied the protein expression of H3K4-3Me, H3K9-3Me, H3K27-3Me, H3K36-3Me, H3K79-3Me, and H3 in A549 lung cancer cells, MDA-MB-231 breast cancer cells, and HepG2 liver cancer cells using immunofluorescence. Our results showed that the protein expression of the H3 methylation markers within the nucleus increases in the aspirin-treated groups versus control (Fig. 4b). To further support this, we extracted the nuclear proteins of each group and detected these H3-3Me markers; the results also showed that the expression of H3-3Me markers was increased within the nucleus in the aspirin-treated groups versus control (Fig. 4c, S3B).

In order to identify the role of H3 methylation in regulating selected inflammation-related stemness genes, we measured the amount of ICAM3 DNA fragments in H3 modification marker pull-downed DNAs in A549 lung cancer cells, MDA-MB-231 breast cancer cells, and HepG2 liver cancer cells using the CHIP-qPCR assay. We selected ICAM3 since our previous studies demonstrated that ICAM mediates cancer cell inflammation and stemness. Our results demonstrated that the amount of ICAM3 DNA fragments in the various H3 methylation marker pull-downed DNAs decreases in all three cancer cell lines (Fig. 4c). The abovementioned findings suggest that aspirin reduces histone demethylase (i.e., KDM6A and KDM6B) expression which mediates histone 3 methylation and thereby inhibits gene expression in vitro.

### Aspirin mediates H3 methylation to regulate ICAM3 expression in vivo

In order to confirm the above in vitro results, we next examined H3 methylation marker expression in tumors from aspirin-treated mice versus control using immunocytochemistry. Our results demonstrated that the H3 methylation marker immunostaining within the nucleus increases in the aspirin-treated group versus control (Fig. 4d, e). We also found that the amount of ICAM3 DNA fragments in the various H3 methylation marker pull-downed DNAs decreases in the aspirin-treated group versus control indicating that ICAM3 expression is blocked (Fig. 4f). These findings suggest that aspirin mediates H3 methylation and thereby regulates ICAM3 expression in vivo.

### Aspirin mediates H3 methylation to regulate ICAM3 expression via a COX-independent manner

**Fig. 5:**
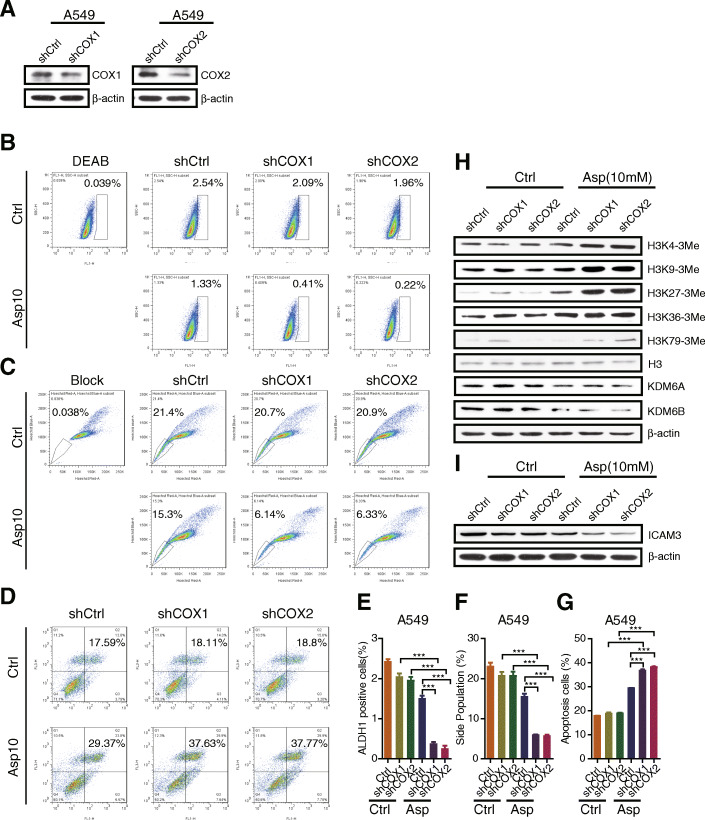
Aspirin mediates H3 methylation to regulate ICAM3 expression via a COX-independent manner. **a** Western blot examining the COX1 and COX2 knockdown efficiency in A549 cells. **b** ALDH staining assay was performed to check ALDH+ sub-population percentage in A549-shCtrl, shCOX cells with or without aspirin treatment. **c** Side population assay was performed to detect SP percentage in A549-shCtrl, shCOX cells with or without aspirin treatment. **d** FACS was performed to detect cell resistance to cisplatin, and the percentage of apoptotic cells was shown. **e**–**g** Statistic results of ALDH+ sub-population, side population, and apoptosis cell percentage were shown. **h** Western blot examining the expression of histone 3 methylation markers, KDM6A, KDM6B in A549-shCtrl, shCOX cells with or without aspirin treatment. **i** Western blot examining the expression of ICAM3 in A549-shCtrl, shCOX cells with or without aspirin treatment

In order to determine the role of COX in aspirin-mediated H3 methylation and targeted gene expression, we knocked down COX1 and COX2 expression in A549 cells, respectively (Fig. 5a, S3C), and examined the ALDH+ population, side population, and chemo-resistance. The results showed that the ALDH+ population (Fig. 5b, e) and side population (Fig. 5c, f) were decreased in shCOX1 or shCOX2 cells treated with aspirin compared to shCOX1 or shCOX2 cells treated with DMSO, and also the ALDH+ population and side population were decreased in shCOX1 or shCOX2 cells treated with aspirin compared to shCtrl treated with aspirin. Moreover, the apoptosis was increased in shCOX1 or shCOX2 cells treated with DDP and aspirin compared to shCOX1 or shCOX2 cells treated with DDP and DMSO ctrl, and also the apoptosis was increased in shCOX1 or shCOX2 cells treated with DDP and aspirin compared to shCtrl treated with DDP and aspirin (Fig. 5d, g). In addition, western blot results displayed that the H3 tri-methylation markers were increased, and the histone demethylases (i.e., KDM6A and KDM6B) were decreased in shCOX1 or shCOX2 cells treated with aspirin compared to shCtrl treated with aspirin (Fig. 5h, S3D). Accordingly, as the new target genes, ICAM3 expression was decreased in shCOX1 or shCOX2 cells treated with aspirin versus shCtrl treated with aspirin (Fig. 5i, S3E). These findings suggest that aspirin mediates H3 methylation and thereby regulates ICAM3 expression via a COX-independent manner.

### Aspirin combined with HDM (KDM6A/B) or ICAM3 signaling inhibitors diminish cancer progression in vivo

**Fig. 6:**
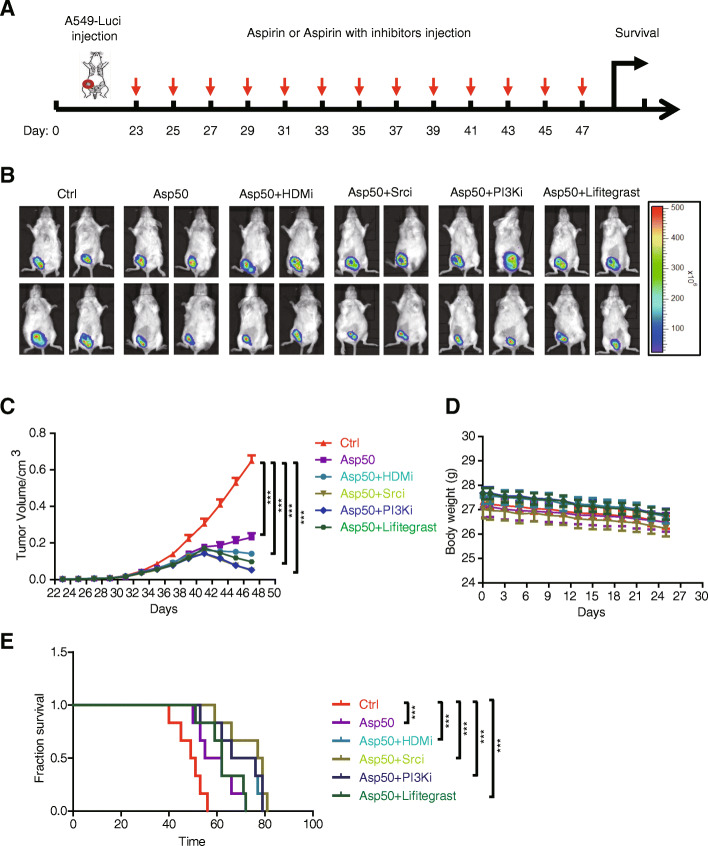
Aspirin combined HDM (KDM6A/B) or ICAM3 signaling inhibitors restrained cancer progression in vivo. **a** Schema of the inhibitor therapy model established by subcutaneous implantation of A549-luci cells into the NOD/SCID mice. **b** The representative luciferase images showing the A549-luci tumors at the primary sites of each group. **c** Tumor growth curve of A549-luci cells under combination therapy of aspirin and HDM inhibitor, Src/PI3K inhibitors, or Lifitigrast. **d** The body weight of BALB/c mice inoculated with A549-luci cells under combination therapy of aspirin and HDM inhibitor, Src/PI3K inhibitors, or Lifitigrast. **e** The survival curve of BALB/c mice inoculated with A549-luci cells under combination therapy of aspirin and HDM inhibitor, Src/PI3K inhibitors, or Lifitigrast

Our previous work proved that ICAM3 could mediate Src/PI3K signaling to promote cancer cell stemness. In order to investigate the use of aspirin combined with HDM (KDM6A/B) or ICAM3 signaling inhibitors as the therapeutic strategies, we implanted A549-luciferase cells into the fourth fat pad of male NOD/SCID mice. Twenty-three days after implantation, we injected IP the mice with 20 mg/kg aspirin, 20 mg/kg aspirin + 100 mg/kg KDM6A/B inhibitor (GSK J1), 20 mg/kg aspirin + 20 mg/kg Src inhibitor, 20 mg/kg aspirin + 70 mg/kg PI3K inhibitor (LY294002), 20 mg/kg aspirin + 20 mg/kg Lifitigrast (ICAM3 inhibitor), or DMSO (control group) every 2 days (Fig. 6a). Our results showed that tumor size and tumor volume decreases in the aspirin-treated group and the aspirin + inhibitor-treated groups versus DMSO control (Fig. 6b, c). However, we found that the body weight did not change significantly in the aspirin-treated group and the aspirin + inhibitor-treated groups versus DMSO control (Fig. 6d).

In addition, we found that the survival time increases in the aspirin-treated group and the aspirin + inhibitor-treated groups versus DMSO control (Fig. 6e). These results suggest that aspirin combined with HDM (KDM6A/B) or ICAM3 signaling inhibitors diminish cancer progression in vivo and may serve as the therapeutic strategies.

### Proposed model of aspirin inhibits cancer cell stemness and cancer progression

**Fig. 7:**
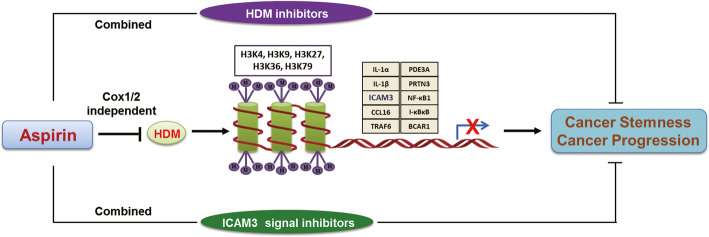
Proposed model of aspirin inhibits cancer cell stemness and cancer progression

Based on our findings, we propose the following model (Fig. 7). Aspirin inhibits histone demethylase (HDM) expression which then mediates histone 3 methylation (H3K4-3Me, H3K9-3Me, H3K27-3Me, H3K36-3Me, H3K79-3Me), respectively. These H3 methylations then inhibit the expression of various inflammation-related stemness genes previously identified by high-throughput siRNA screening (IL-1a, IL-1B, ICAM3, CCL16, TRAF6, PDE3A, PRTN3, NF-kB1, I-kBkB, BCAR1). Using the ICAM3 gene as a representative of the inflammation-related stemness genes, by the aspirin-mediated H3 modifications restrain ICAM3 promoter activity and cause ICAM3 expression is inhibited. Thus, aspirin may diminish cancer cell stemness properties and cancer progression in vitro and in vivo by inhibiting the expression of various inflammation-related stemness genes. Most interestingly, the above process was not depending on COX expression. As the therapeutic strategies, aspirin combined various inhibitors suppressed tumor progression effectively.

## Discussion

The widely recognized anti-cancer potential of aspirin (a classical non-steroidal and anti-inflammatory drug) has created a broad interest to explore the clinical benefits of aspirin in cancer therapy [29–31]. Previous findings by many investigators have established that aspirin induces apoptosis in cancer cells and inhibits proliferation and metastasis of cancer cells. In addition, aspirin inhibits cancer stemness in esophageal cancer and colorectal cancer although the mechanism of action remains unclear [16, 32–35]. In this study, we investigated the role of aspirin on cancer stemness in breast cancer, lung cancer, and liver cancer. We found that aspirin diminishes cancer cell stemness properties which include reducing the ALDH+ subpopulation, side population, chemo-resistance, and sphere formation in all three cancer types in vitro. Also, aspirin inhibits tumor growth, metastasis, and chemo-resistance and prolongs survival in vivo. Our in vitro and in vivo studies reveal that the inhibitory role of aspirin occurs on multiple fronts in all three cancer types.

The well-characterized mechanism of action for aspirin involves the modification of Cox enzymes [36]. In regards to aspirin in cancer therapy, early reports focused on the inhibition of the Cox-dependent pathway which leads to reduced inflammation and hence the anti-cancer properties of aspirin. Besides the inhibition of the Cox-dependent pathway, it is now clear that other pathways and multiple molecular mechanisms play a role in the anti-cancer properties of aspirin [32]. In this regard, aspirin inhibits HDACs expression which effects histone modification in colorectal cancer and other cell types [16, 21]. In our study, we performed a more detailed analysis of the action of aspirin on histone methylation. Our findings indicate that aspirin reduces histone demethylase (KDM6A/B) expression which mediates histone 3 methylation and thereby suppresses inflammation-related stemness gene expression. These findings suggest a novel molecular mechanism that explains the anti-cancer properties of aspirin.

Cancer stem cells (CSCs) are a small population of cancer cells that possess the ability to self-renew, differentiate, and modulate cancer growth, recurrence, metastasis, and chemo-resistance [3, 8, 37, 38]. The maintenance of cancer cell stemness largely depends on the surrounding inflammatory microenvironment [39]. Our previous work established a medium-throughput siRNA screening platform to identify inflammation genes that regulate cancer cell stemness and identified several novel candidates (e.g., ICAM3). ICAM3 mediates cancer cell stemness as well as cancer-related inflammation via Src/PI3K/AKT signaling [8]. In our study, we clearly demonstrated that aspirin inhibits the expression of inflammation-related stemness genes (especially ICAM3). Moreover, aspirin mediates histone methylation which causes an inhibition of inflammation-related stemness gene transcription which further suppresses cancer stemness. Our findings identify novel targets of aspirin that may explain the anti-cancer properties of aspirin and may lead to new therapeutic strategies. In conclusion, our results demonstrated that aspirin diminishes cancer cell stemness properties and cancer progression in vitro and in vivo. Moreover, aspirin inhibits inflammation-related stemness gene expression especially ICAM3 that we screened by high throughput siRNA platform.

Our investigation of the underlying molecular mechanism demonstrated that aspirin reduces histone demethylase (KDM6A/B) expression. This reduction mediates histone 3 methylation and thereby inhibits inflammation-related stemness gene expression. In regards to therapeutic strategies, aspirin combined with HDM inhibitors, Src/PI3K inhibitors, or ICAM3 inhibitor Lifitigrast diminished cancer progression in vivo. Therefore, our findings reveal a novel molecular mechanism that sheds further light on the anti-cancer properties of aspirin and suggest therapeutic strategies for the prevention of cancer progression.

## Conclusions

In conclusion, we disclosed that aspirin diminishes cancer cell stemness properties in vitro*.* We reported that aspirin inhibits tumor growth and metastasis and prolongs survival in vivo. We proved that aspirin inhibited inflammation-related stemness gene expression especially ICAM3 that we screened by high-throughput siRNA platform. We demonstrated that aspirin reduced histone demethylase (KDM6A/B) expression to mediate histone 3 methylation to suppress gene expression via a COX-independent manner. We used aspirin combined HDM (KDM6A/B) inhibitors or ICAM3 inhibitor Lifitigrast or ICAM3 downstream signaling Src/PI3K inhibitors restrained cancer progression in vivo.

## Supplementary information

**Additional file 1: Figure S1.** The work concentration of aspirin was tested in various cancer cells. **Figure S2.** Quantification results of western blot data. **Figure S3.** Quantification results of western blot data. **Table S1.** Antibodies List. **Table S2.** Primer sequences.

## Data Availability

The data used and analyzed during this study are available from the corresponding author on request.

## Abbreviations

ICAM3: Intercellular adhesion molecule 3
HDM: Histone demethylase
COX: Cyclooxygenase
KDM6A/B: Lysine demethylase 6A/B
PDE3A: Phosphodiesterase 3A
PRTN3: Proteinase 3
