# Supplementary material for: Aspirin mediates histone methylation that inhibits inflammation-related stemness gene expression to diminish cancer stemness via COX-independent manner
Source: Stem Cell Res Ther. 2020 Aug 27;11:370. doi: 10.1186/s13287-020-01884-4 (PMC7450956; doi:10.1186/s13287-020-01884-4)
Supplement: Supplementary file 1 — Additional file 1: Figure S1. The work concentration of aspirin was tested in various cancer cells. Figure S2. Quantification results of western blot data. Figure S3. Quantification results of western blot data. Table S1. Antibodies List. Table S2. Primer sequences. [file 13287_2020_1884_MOESM1_ESM.pdf]

Figure S1

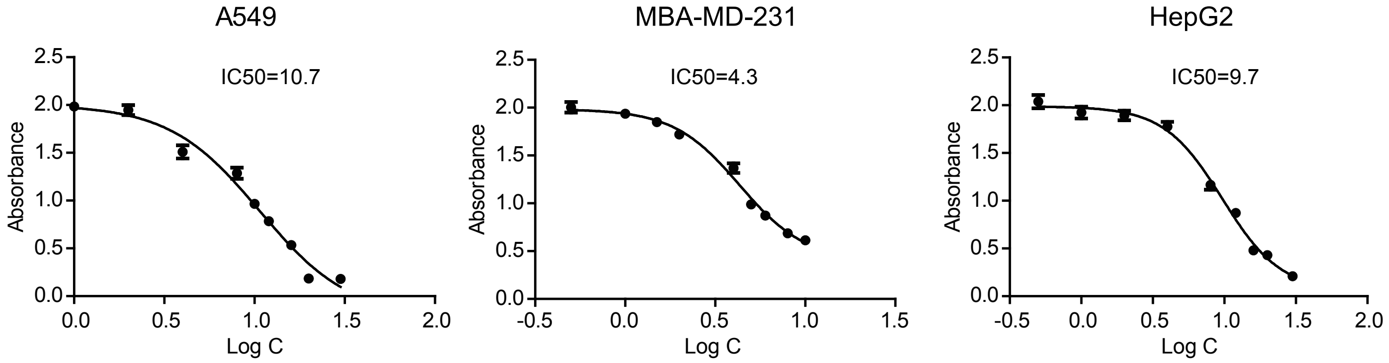

A549
IC50=10.7
Absorbance
Log C
MBA-MD-231
IC50=4.3
Absorbance
Log C
HepG2
IC50=9.7
Absorbance
Log C

Fig. S1 The work concentration of aspirin was tested in various cancer cells.

The cytotoxicity assay was performed on breast cancer cell lung cancer cell A549, MDA-MB-231and liver cancer cell HepG2. The IC50 of aspirin on three cell lines were 10.7, 4.3, 9.7 respectively.

Figure S2

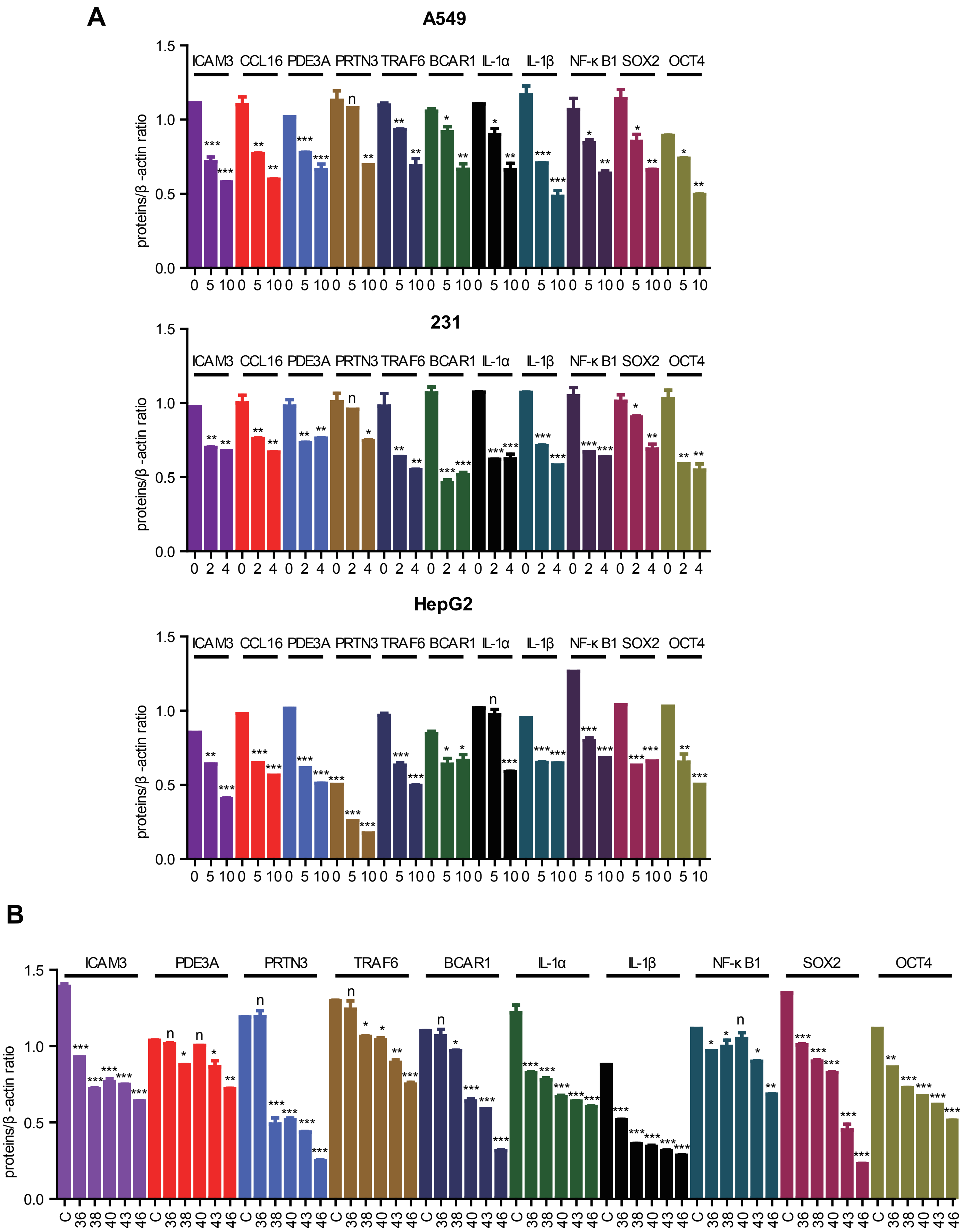
A
A549
231
HepG2
proteins/β -actin ratio
ICAM3 CCL16 PDE3A PRTN3 TRAF6 BCAR1 IL-1α IL-1β NF-κ B1 SOX2 OCT4
0 5 100 5 100 5 100 5 100 5 100 5 100 5 100 5 100 5 100 5 100 5 10
0 2 4 0 2 4 0 2 4 0 2 4 0 2 4 0 2 4 0 2 4 0 2 4 0 2 4 0 2 4 0 2 4
B
ICAM3 PDE3A PRTN3 TRAF6 BCAR1 IL-1α IL-1β NF-κ B1 SOX2 OCT4
C 36 38 40 43 46
0.0 0.5 1.0 1.5

Fig. S2 Quantification results of the western blot data by three biological repeats. (A) Quantification results of figure 3B. (B) Quantification results of figure 3F. *Indicates significant difference with $P<0.05$, **indicates significant difference with $P<0.01$, ***indicates significant difference with $P<0.001$.

Figure S3

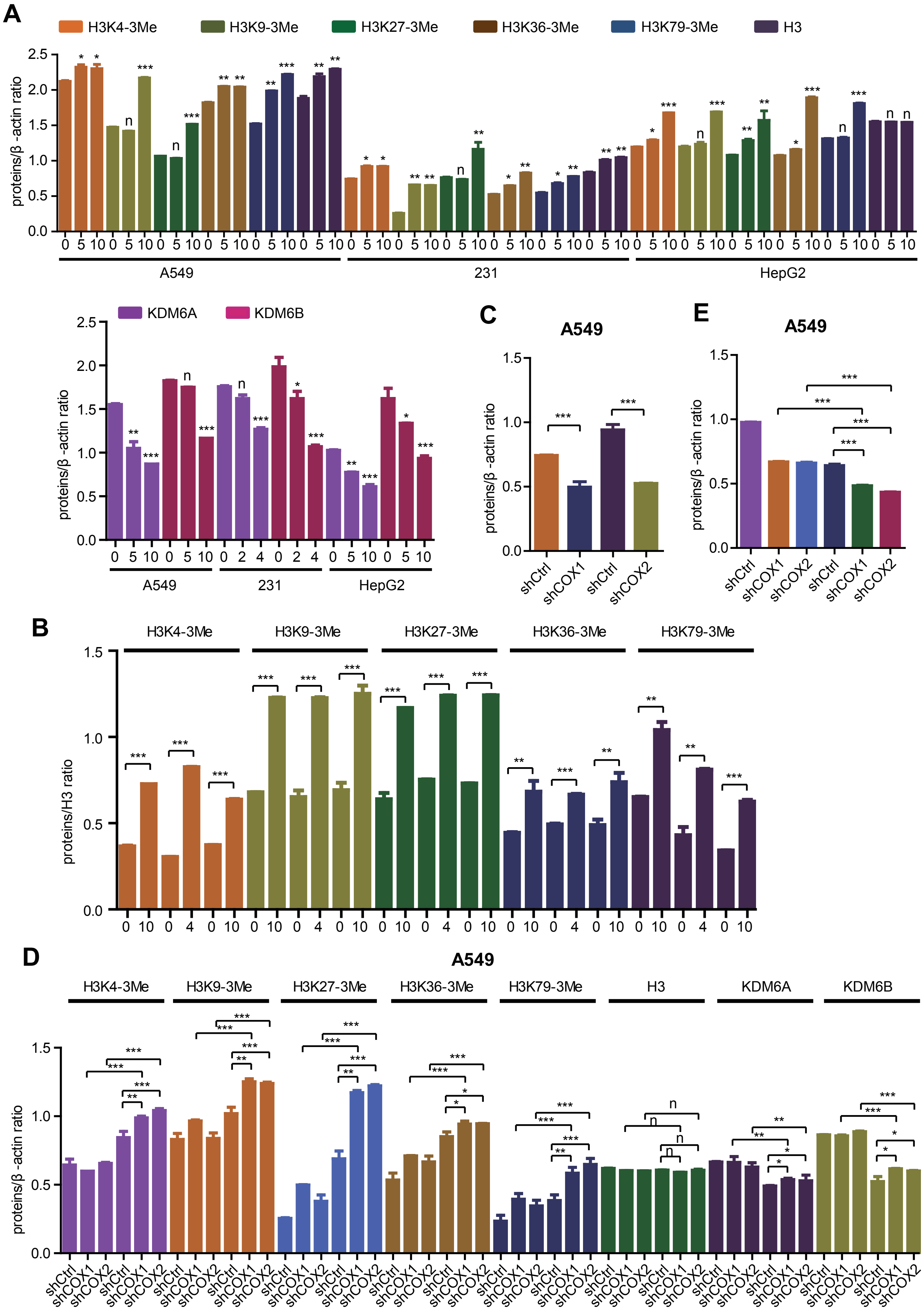
A
H3K4-3Me
H3K9-3Me
H3K27-3Me
H3K36-3Me
H3K79-3Me
H3
proteins/β -actin ratio
A549
231
HepG2
KDM6A
KDM6B
C
A549
shCtrl
shCOX1
shCtrl
shCOX2
E
A549
shCtrl
shCOX1
shCOX2
shCtrl
shCOX1
shCOX2
B
H3K4-3Me
H3K9-3Me
H3K27-3Me
H3K36-3Me
H3K79-3Me
proteins/H3 ratio
D
A549
H3K4-3Me
H3K9-3Me
H3K27-3Me
H3K36-3Me
H3K79-3Me
H3
KDM6A
KDM6B
proteins/β -actin ratio

Fig. S3 Quantification results of the western blot data by three biological repeats. (A) Quantification results of figure 4A. (B) Quantification results of figure 4C. (C) Quantification results of figure 5A. (D) Quantification results of figure 5H. (E) Quantification results of figure 5I. *Indicates significant difference with $P<0.05$, **indicates significant difference with $P<0.01$, ***indicates significant difference with $P<0.001$.

Supplemental Table 1. Antibodies List

Antibody		Clone, Cat #	Vendor	City, State, Country
ICAM3	Rabbit monoclonal	EPR3994, ab109405	Abcam	Hong Kong, China
CCL16	Rabbit monoclonal	EPR4452(2), ab134917	Abcam	Hong Kong, China
PDE3A	Rabbit monoclonal	EPR11601, ab169534	Abcam	Hong Kong, China
PRTN3	Rabbit monoclonal	EPR6227, ab133613	Abcam	Hong Kong, China
IL-1α	Rabbit polyclonal	ab9614	Abcam	Hong Kong, China
OCT4	Rabbit polyclonal	ab19857	Abcam	Hong Kong, China
SOX2	Rabbit polyclonal	H-65, sc-20088X	Santa Cruz Biotechnology	Santa Cruz, CA, USA
TRAF6	Rabbit polyclonal	H-274, sc-7221	Santa Cruz Biotechnology	Santa Cruz, CA, USA
BCAR1	Rabbit polyclonal	C-20, sc-860	Santa Cruz Biotechnology	Santa Cruz, CA, USA
β-actin	Mouse monoclonal	sc-47778	Santa Cruz Biotechnology	Santa Cruz, CA, USA
IL-1β	Mouse monoclonal	12242	Cell Signal Technology	Danvers, MA, USA
p-IKKβ	Mouse monoclonal	16A6, 2697L	Cell Signal Technology	Danvers, MA, USA
H3K4-3Me	Rabbit monoclonal	9783 (Kit)	Cell Signal Technology	Danvers, MA, USA
H3K9-3Me	Rabbit monoclonal	9783 (Kit)	Cell Signal Technology	Danvers, MA, USA
H3K27-3Me	Rabbit monoclonal	9783 (Kit)	Cell Signal Technology	Danvers, MA, USA
H3K36-3Me	Rabbit monoclonal	9783 (Kit)	Cell Signal Technology	Danvers, MA, USA
H3K79-3Me	Rabbit monoclonal	9783 (Kit)	Cell Signal Technology	Danvers, MA, USA
H3	Rabbit monoclonal	9783 (Kit)	Cell Signal Technology	Danvers, MA, USA
KDM6A	Rabbit monoclonal	33510	Cell Signal Technology	Danvers, MA, USA
KDM6B	Rabbit monoclonal	3457	Cell Signal Technology	Danvers, MA, USA

Supplemental Table 2. Primer sequences

Name	Sequence
ICAM3-H-RT-F	CCCCAGCACTTGAAATGGAAAGA
ICAM3-H-RT-R	AGGGTCAGTAACACCGCCACGAA
CCL16-RT-F	CTTATCATTACTTCGGCTTCTCGC
CCL16-RT-R	GGCCTTTCTGTATCCCACCACTA
PDE3A-RT-F	GATGATAAATACGGATGTCTGTCTGG
PDE3A -RT-R	GACAAGGAAACGGAAATGCTTAA
PRTN3-RT-F	ACAACTACGACGCGGAGAACAAACTGA
PRTN3-RT-R	GAAGAAGGTGACCACGGTGACATTGAG
TRAF6-RT-F	GCACGCCACCTACAAGAG
TRAF6-RT-R	CAGGGCTATGAATCACAACA
BCAR1-RT-F	CAGTTTGAACGACTGGAACAGGAGGTG
BCAR1-RT-R	AGCTTGTGGGCGCTGAGGATGAC
IL-1α-RT-F	ATTGTATGTGACTGCCCAAGATG
IL-1α-RT-R	GTTTCCCAGAAGAAGAGGAGGTT
IL-1β-RT-F	GGCGGCATCCAGCTACGAATCTC
IL-1β-RT-R	AAGGTCTGTGGGCAGGGAACCAG
NF-κB1-RT-F	AAAAGGACCCTGAAGGTTGTGAC
NF-κB1-RT-R	AAGGTGGATGATTGCTAAGTGTAAG

IκBκB-RT-F	GGCAGTCTTTGCACATCATTCGT
IκBκB-RT-R	TCACCGTTCCATTCAAGTCTTCG
SOX2-RT-F	TGGAGCAACGGCAGCTACAGCATG
SOX2-RT-R	GGAGTGGGAGGAAGAGGTAACCACAGG
OCT4-RT-F	TGGGAAGGTATTCAGCCAAACGA
OCT4-RT-R	ACCGAGGAGTACAGTGCAGTGAAGTGA
ICAM3-M-RT-F	GCAGAACAGGAAGGCACCAAACAG
ICAM3-M-RT-R	CTCAAAGTCAGAAGAGGAGTCGGGAAG
ICAM3-CHIP-3-F	CTTGCACAGGAACAGTAGCG
ICAM3-CHIP-3-R	ACGAAGAACGGGATCCC
